# The Diagnostic Value of Fine-Needle Aspiration Cytology in Cervical Lymphadenopathy in Correlation to Postoperative Histopathological Results in a Tertiary Care Center in Saudi Arabia

**DOI:** 10.7759/cureus.46210

**Published:** 2023-09-29

**Authors:** Maraam M Al Qout, Mohammed Al Hamoud, Mubarak S AlQahtani, Alhanouf Y Alqahtani, Abdullah H Asiri, Abdulrahman A Alshahrani

**Affiliations:** 1 College of Medicine, King Khalid University, Abha, SAU; 2 Department of Otorhinolaryngology - Head and Neck Surgery, Aseer Central Hospital, Abha, SAU

**Keywords:** fine-needle aspiration cytology (fnac), diagnostic value, histological diagnosis, tru-cut biopsy, cervical lymphadenopathy

## Abstract

Background

Lymphadenopathy is a frequently encountered presentation in the clinical practice. Cervical lymphadenopathy implies that the cervical nodal tissue measures more than 1 cm in diameter. It requires prompt and accurate diagnosis to begin an appropriate treatment plan. Fine-needle aspiration cytology (FNAC) is considered an initial diagnostic method due to its simplicity, minimal invasiveness, quick availability of results, and low risk of complications. This study aimed to evaluate the diagnostic value of FNAC by comparing the cytological and histological diagnoses of patients with cervical lymph node enlargement at Aseer Central Hospital, Southern Region, Saudi Arabia.

Methodology

This observational, retrospective, record review study was conducted at the Otorhinolaryngology Head and Neck Surgery Department in Aseer Central Hospital, Abha, Saudi Arabia. Using a data collection sheet, the data of 102 patients were collected from electronic records and reviewed retrospectively. The study included patients who underwent cervical lymph node excision biopsy between 2020 and 2023 due to enlargement of the cervical lymph node. The cytological diagnoses were compared with the histopathological diagnoses of the same enlarged cervical lymph nodes.

Results

The most common FNAC findings were lymphomas and reactive lymph nodes (26.2% and 19.7%, respectively). The positive predictive value of FNAC was 100% and the negative predictive value was 86.7%. Overall, the diagnostic accuracy was 95.3%.

Conclusions

FNAC is a safe diagnostic method with minimal invasiveness and complications. This study showed that FNAC and tru-cut biopsy have good diagnostic value in examining patients with cervical lymphadenopathy regardless of their limitations and drawbacks. They have good sensitivity, specificity, positive and negative predictive values, and accuracy.

## Introduction

Lymphadenopathy is a common clinical presentation seen by physicians. It can present as abnormal enlargement or alterations in the number or consistency of the lymph nodes [1]. Cervical lymphadenopathy (CLA) indicates that the cervical nodal tissue is enlarged by more than 1 cm in diameter. Its presence is a sign of an underlying local or systemic pathological condition, which includes infections, medications, autoimmune diseases, and malignancies [2]. A study investigating the etiology of CLA found that the most common cause varied according to the age group. Tuberculosis (TB) was the main cause among patients between 14 and 59 years old, while cancer predominated in individuals who were 60 years old or above [3].

A thorough history and physical examination should be implemented in diagnosing CLA; however, clinical assessment alone is not enough because the patient’s presentation can mimic other conditions [4]. Fine-needle aspiration cytology (FNAC) is the initial step in diagnosing lymphadenopathy due to its simplicity, rapid availability of results, and minimal complications [5]. According to the guidelines of the American Academy of Otolaryngology-Head and Neck Surgery, FNAC should be performed, rather than open biopsy, for patients with an increased risk of malignancy [6]. As open biopsy is associated with non-healing wounds, localized reoccurrence, and distant metastasis, performing open biopsy as an initial diagnostic tool for neck masses should be avoided as much as possible [7].

Several studies have revealed an increase in the diagnostic accuracy of FNAC due to the evolving immunohistochemical analytical methods [8-10]. A meta-analysis aimed to assess the effectiveness of FNAC in investigating head and neck masses regardless of their specific location. It revealed that FNAC had an overall accuracy of 93.1%. The overall sensitivity, specificity, positive predictive value (PPV), and negative predictive value (NPV) were 89.6%, 96.5%, 96.2%, and 90.3%, respectively [11]. Despite the presence of a wide variety of methods that aid in diagnosing lymphadenopathy, excisional biopsy remains the definitive diagnostic method due to the maintained lymph nodal structure in the specimen [12].

According to Attard et al., FNAC is an excellent tool to diagnose metastatic malignancy, which reduces the need to use excisional biopsy. However, the limitation was in differentiating between types of reactive lymphoid hyperplasia and low-grade non-Hodgkin lymphoma [13]. The use of FNAC in diagnosing CLA remains controversial and is mostly followed by excisional biopsy. Hence, this study aims to evaluate the diagnostic value of FNAC by comparing the cytological and histological diagnoses of patients with cervical lymph node enlargement at Aseer Central Hospital, Southern Region, Saudi Arabia.

## Materials and methods

This observational, retrospective, record review study was performed on 102 patients at the Otorhinolaryngology Head and Neck Surgery Department at Aseer Central Hospital, Abha, Saudi Arabia. Patients who underwent cervical lymph node excisional biopsy due to cervical lymph node enlargement between 2020 and 2023 were included and examined retrospectively. The data of the patients were collected from electronic records.

Inclusion criteria

Patients who underwent cervical lymph node excisional biopsy between 2020 and 2023 were included. Patients who underwent FNAC or tru-cut biopsy and postoperative excision biopsy were considered for inclusion. The participants were adolescents and adults of both genders and of all races and ethnicities.

Exclusion criteria

Patients who underwent lymph node excisional biopsy of lymph nodes other than cervical lymph nodes were excluded. Moreover, patients with CLA who underwent postoperative excision biopsy without either preoperative FNAC or tru-cut biopsy were excluded.

Data collection

Based on a literature review, a standard data collection sheet was developed for this study to avoid errors during data extraction. The sheet was sent to two expert otorhinolaryngology surgeons from Aseer Central Hospital to check its content validity and clarity. Data that were extracted included patients’ age, gender, preoperative histopathological study performed (FNAC, tru-cut biopsy, or both), FNAC results, tru-cut biopsy results, and postoperative histopathological results. The results were either reactive lymph node, lymphoma, TB, suspicious of malignancy, malignancy, and unsatisfactory.

Data analysis

In this study, the results of the cytological examination were classified into five categories, namely, reactive lymph node, TB, lymphoma, malignancy, and suspected malignancy. The outcomes were further coded on a dichotomous scale to be easily incorporated into the diagnostic performance analysis. The dichotomous scale included benign lesions (reactive lymph node and TB) and malignant lesions (lymphoma, malignancy, and suspected malignancy). Inconclusive cases were considered unsatisfactory and were not included in the analysis of diagnostic performance. The sensitivity was computed based on malignant cases, whereas specificity was based on benign cases. Results of the diagnostic performance were presented along with their respective 95% confidence intervals (95% CIs). Statistical analysis was performed using RStudio (R version 4.3.0).

## Results

**Figure 1:**
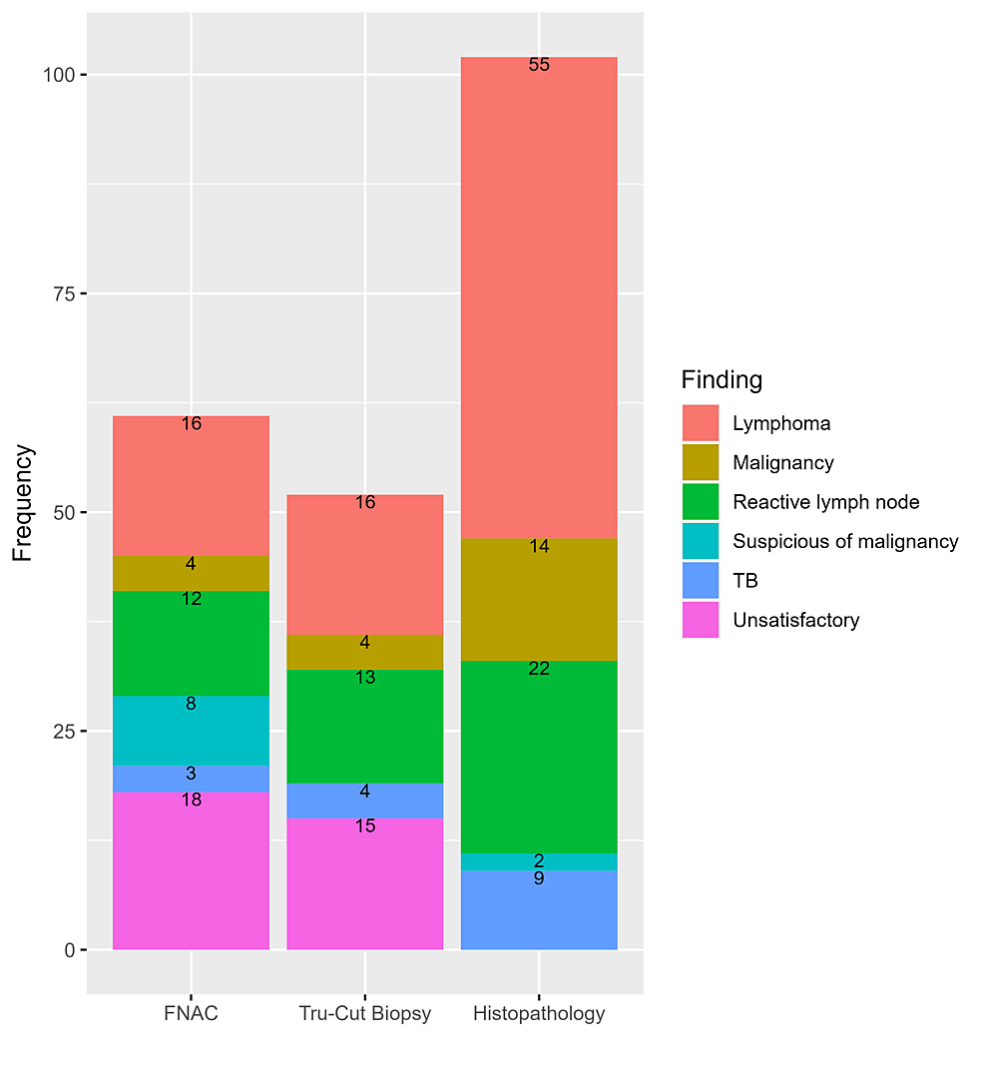
The frequency of findings indicated by different diagnostic methods used in this study. FNAC: fine-needle aspiration cytology; TB: tuberculosis

In this study, we analyzed data from 102 patients with available histopathological findings. Fewer than one-third of patients (29.4%) were aged 20 to 30 years, and more than half were males (57.8%) (Table 1). Of the included patients, the results of FNAC were available for 61 patients, and tru-cut biopsy results were available for 52 patients. The distributions of FNAC and tru-cut biopsy findings are depicted in Figure 1.

**Table 1:** Demographic characteristics of patients (n = 102).

Parameter	Category	N (%)
Age	Less than 20 years	18 (17.6%)
	20–30 years	30 (29.4%)
	31–40 years	13 (12.7%)
	41–50 years	18 (17.6%)
	More than 50 years	23 (22.5%)
Gender	Male	59 (57.8%)
	Female	43 (42.2%)

Results of the FNAC and histopathology analyses

The most common findings of the FNAC analysis were lymphomas (26.2%) and reactive lymph nodes (19.7%). Likewise, lymphomas and reactive lymph nodes were frequent in tru-cut biopsy (30.8% and 25.0%, respectively) and postoperative histopathological analyses (53.9% and 21.6%, respectively) (Table 2).

**Table 2:** Results of the histopathology, FNAC, and tru-cut biopsy analyses. FNAC: fine-needle aspiration cytology

Characteristic	Histopathology (n = 102)	FNAC (n = 61)	Tru-cut biopsy (n = 52)
Reactive lymph node	22 (21.6%)	12 (19.7%)	13 (25.0%)
Tuberculosis	9 (8.8%)	3 (4.9%)	4 (7.7%)
Lymphoma	55 (53.9%)	16 (26.2%)	16 (30.8%)
Malignancy	14 (13.7%)	4 (6.6%)	4 (7.7%)
Suspicious of malignancy	2 (2.0%)	8 (13.1%)	0 (0.0%)
Unsatisfactory	0 (0.0%)	18 (29.5%)	15 (28.8%)

Inconclusive cases

Notably, more than one-quarter of FNAC and tru-cut biopsy findings were unsatisfactory (29.5% and 28.8%, respectively) (Table 2). These unsatisfactory cases were excluded from the subsequent analyses of the diagnostic accuracy testing.

Analysis of the diagnostic accuracy of FNAC

Based on 43 samples with satisfactory records, 30 (69.8%) cases were malignant and 13 (30.2%) cases were benign on the postoperative histopathological examination. A total of 28 cases (out of 30 records) showed evidence of malignancy or suspicious malignancy on FNAC, with a sensitivity of 93.3%. Two cases were falsely detected as benign cases (6.7% were false negative). On the other hand, all benign cases on histopathology were detected by FNAC (specificity was 100%). In general, FNAC had a PPV of 100% and an NPV of 86.7%. Overall, the diagnostic accuracy was 95.3% (Table 3).

**Table 3:** Analysis of the diagnostic performance of FNAC against postoperative histopathological findings (N = 43). Results are expressed as values (95% confidence intervals). FNAC: fine-needle aspiration cytology; PPV: positive predictive value; NPV: negative predictive value

Parameter	Category	Histopathology	
		Malignant (N = 30)	Benign (N = 13)
FNAC	Malignant/Suspicious	28 (93.3%)	0 (0.0%)
	Benign	2 (6.7%)	13 (100.0%)
Diagnostic testing*	Sensitivity	93.3 (77.9-99.2)	
	Specificity	100 (75.3-100)	
	PPV	100 (87.7-100)	
	NPV	86.7 (59.5-98.3)	
	Diagnostic accuracy	95.3 (84.2-99.4)	

Analysis of the diagnostic accuracy of tru-cut biopsy

Based on 37 samples with satisfactory records in tru-cut biopsy, an aggregate of 20 patients (constituting 90.9% of the total 22 patients) exhibited indications of malignancy or suspicion of malignancy upon tru-cut biopsy assessment. This was accompanied by a meager count of two instances erroneously identified as negative for malignancy (thus, a 9.1% misclassification as benign). In contrast, all benign cases in the histopathological examination were successfully detected by tru-cut biopsy, for a specificity rate of 100%. Tru-cut biopsy displayed a PPV of 100% and an NPV of 88.2%. Overall, the diagnostic accuracy was 94.6% (Table 4).

**Table 4:** The diagnostic performance of tru-cut biopsy against postoperative histopathological findings (n = 37). PPV: positive predictive value; NPV: negative predictive value

Parameter	Category	Histopathology	
		Malignant (N = 22)	Benign (N = 15)
Tru-cut biopsy	Malignant/Suspicious	20 (90.9%)	0 (0.0%)
	Benign	2 (9.1%)	15 (100.0%)
Diagnostic testing*	Sensitivity	90.9 (70.8-98.9)	
	Specificity	100 (78.2-100)	
	PPV	100 (83.2-100)	
	NPV	88.2 (63.6-98.5)	
	Diagnostic accuracy	94.6 (81.8-99.3)	

## Discussion

CLA is a frequently occurring clinical condition that must be promptly and accurately diagnosed to begin an appropriate treatment plan as soon as possible. FNAC is often used in the preliminary assessment of enlarged lymph nodes, as it is quick, affordable, minimally invasive, reliable, and practical. Early FNAC can guide additional testing and evaluation, reduce patient anxiety, and save time, money, and patient morbidity [14]. This study compares the cytological and histological diagnoses of individuals with enlarged cervical lymph nodes to assess the diagnostic usefulness of FNAC and true-cut biopsy. FNAC showed good validity and performance, with a sensitivity of 93.5%, a specificity of 100%, a PPV of 100%, and an NPV of 86.7%. The accuracy of the test was 86.7%. Tru-cut biopsy also had high diagnostic performance and validity, with a sensitivity of 90.9%, a specificity of 100.0%, a PPV of 100%, and an NPV of 88.2%. The accuracy of the test was 94.6%.

Lesions originating within the lymph nodes can manifest in individuals of diverse age groups. In our study, a relatively even distribution was observed across each decade, with the exception of the 21-30-year age group, which accounted for 19.4% of presentations. In contrast, the age group of 31-40 years constituted 12.7% of cases. Regarding sex, more cases were reported among males compared to females (57.8% vs. 42.2%).

The majority of the cases in the study involved malignant lymphoma (55.0%). Similarly, Hafez et al. [15] reported that 50% of the examined cases revealed lymphomas. This observation was significantly greater than that made by Serrano Egea et al. [16], who reported that 4.5% of the cases in their study were malignant lymphomas. They attributed the reduced lymphoma incidence to the fact that the majority of the trial participants were children, which had a higher percentage of non-specific infections. Moreover, 9.5% of the cases described by Serrano Egea et al. [16] were lymphomas, which is a lower incidence. In their study, Rakhshan and Rakhshan [17] also observed a 22.4% incidence of malignant lymphoma. The fact that we only examined suspect cases and conducted the study in a cancer institute where most of the referred patients had lymphoma may be two reasons for the high percentage of lymphoma in our study.

Reactive lymphadenopathy was the second most frequent diagnosis after lymphoma (55%) with an incidence of 22%. The primary criticisms of FNAC for lymphoma diagnosis center on its inability to accurately subclassify or grade some lymphoma subtypes because it does not provide an architecture evaluation [18]. Clinical and epidemiological information, patient age, symptoms, number and location of lymph nodes, laboratory findings, peripheral blood smear, and other details were used to diagnose reactive lymphadenopathy. Highly cellular smears, polymorphic patterns of cells without malignant characteristics, and a sizable number of tangible entities were among the cytomorphological criteria. However, it was the diagnosis in several studies ranging from 18.9% to 86.4% [19-21].

In this study, FNAC yielded high sensitivity and specificity with a diagnostic accuracy of 86.7%. Likewise, in the literature, the accuracy rate of lymph node FNAC ranges from 82% to 94.4% [13,15,22,23]. FNAC examination revealed 26.2% lymphoma and 19.7% reactive lymph nodes, while around 30% of cases were inconclusive. Similar to our finding, the prevalence of insufficient or inadequate samples varied in the research, ranging from 0% to 25% [14,24]. Due to pervasive crush artifacts and hypocellularity, insufficient specimens were an example of cytological detail that could not be assessed. The small size or type of lesion, such as cystic degeneration, necrosis, or fibrosis, were also considered adequate explanations. In addition, improper handling of the aspirate and a lack of qualified cytopathologists may lead to unsatisfactory aspirates.

As opposed to mere cells obtained with FNAC, a tru-cut biopsy needle can provide a core tissue sample. This can undoubtedly offer a more certain and precise diagnosis. In the head and neck region, benign and malignant soft tissue tumors, thyroid, lymph nodes, and salivary gland lesions are the most common indications for the use of a tru-cut biopsy [24]. It had a high level of sensitivity and specificity with an accuracy of 94.6%. Almost 29.0% of the cases were unsatisfactory. Diagnosed cases were reactive lymph node at 13 (25.0%), TB at four (7.7%), lymphoma at 16 (30.8%), and malignancy at four (7.7%). Another previous study reported that tru-cut biopsy had a sensitivity of 68.42%, a specificity of 83.33%, a PPV of 92.86%, an NPV of 45.45%, and an accuracy rate of 72% [4].

Study strengths and limitations

This study offers valuable information on the diagnostic role of FNAC and tru-cut biopsy in CLA. However, several limitations are worth noting. First, our study was conducted at a single center. Second, the sample size included was relatively small. Subsequent research should aim to encompass a larger and more diverse sample size to yield more definitive and conclusive findings.

## Conclusions

FNAC and tru-cut biopsy appear to have good diagnostic value for examining patients with CLA despite their drawbacks and difficulties. They had good sensitivity, specificity, PPV, and NPV with high accuracy. Proficient cytopathologists should interpret the results of FNAC and tru-cut biopsy in the context of their clinical, radiographic, and laboratory data. To resolve uncertainties regarding these findings, further investigation is necessary to rectify potentially misdiagnosed cases.
